# 39.2 VIRAL EXPOSURES AND SCHIZOPHRENIA

**DOI:** 10.1093/schbul/sby014.160

**Published:** 2018-04-01

**Authors:** Faith Dickerson, Robert Yolken

**Affiliations:** 1 Sheppard Pratt; 2 Johns Hopkins University School of Medicine

## Abstract

**Background:**

Epidemiological, immunological, and microbiological studies indicate that infections with members of the family Herpesviridae may be associated with schizophrenia and with cognitive impairment. Herpesviruses are enveloped, double-stranded DNA viruses which are widely prevalent and which are capable of causing persistent infections. The most highly replicated association is that between the alpha herpesvirus Herpes Simplex Virus Type 1 (HSV-1) and cognitive impairment in schizophrenia. Acute HSV-1 infection results in oral lesions which usually resolve spontaneously. However, latency can occur in nerve root ganglia leading to cycles of reactivation in later life. Other herepsviruses may also be associated with schizophrenia. Epstein Barr virus (EBV) is a gamma herpesvirus usually acquired in childhood or adolescence. Acute EBV infection is often associated with fever and adenopathy leading to a vigorous immune response and the suppression of viral replication. However, latency can occur with long term consequences to the infected individual.

**Methods:**

We examined the association between HSV-1 seropositivity and cognitive functioning in 828 individuals with schizophrenia from the Sheppard Pratt cohort and 573 control individuals. We also studied antibodies to EBV in a recently enrolled subset of the Sheppard Pratt cohort consisting of 397 individuals with schizophrenia and 289 without a psychiatric disorder. Antibodies to HSV-1 and EBV proteins were measured by immunoassay and confirmed by Western blot. Cognitive functioning was measured by the Repeatable Battery for the Assessment of Neuropsychological Status (RBANS). Regression models were employed to define the independent association between virus exposure and outcome.

**Results:**

Serological evidence of exposure to HSV-1 was associated with significantly lower levels of cognitive functioning as measured by the RBANS Total score (coefficient =-3.84, 95% CI -5.60, -2.09, p<.0001). The strongest association was in the domain of Immediate Memory (coefficient= -4.95, 95% CI -7.24, -2.66, p<.0001.) There was a smaller but statistical significant relationship between serological evidence of exposure to HSV-1 and RBANS Total score in control individuals. (coefficient =-1.98, 95% CI -3.88, -.094, p=.04).

In terms of EBV, we found that individuals with schizophrenia had significantly higher levels of antibodies to the EBV viral capsid antigens (VCA) as compared to controls (coefficient= .57, 95% CI .37- .77, p<1.7 10-8). On the other hand, the level of antibody to the EBV Nuclear Antigen (EBNA) and EBV Early Antigen (EA) did not differ between the groups. Within the schizophrenia group, increased levels of EBV VCA antibodies were associated with older age, female gender, and cigarette smoking but not with clinical or cognitive measures.

**Discussion:**

The mechanism of the association between HSV-1 exposure and cognitive deficits in individuals with schizophrenia may be due to underlying neuroanatomical deficits, immune dysregulation, or gene x environmental interactions. The aberrant immune response to EBV may represent underlying immunopathy or exposure to variant viral strains. Other herpesviruses, such as Cytomegalovirus, Herpes Simplex Virus Type 2 and Human Herpesvirus Type 6 have also been associated with schizophrenia risk or with cognitive impairment in some populations. The prevention and treatment of herpesvirus infections might lead to new therapeutic modalities for schizophrenia.

